# Statistical comments on “no seasonal variation in physical activity of Han Chinese living in Beijing”

**DOI:** 10.1186/s12966-017-0604-x

**Published:** 2017-11-03

**Authors:** Mehrdad Farrokhi, Nasser Shirian

**Affiliations:** 1 Medical Student, School of Medicine, Isfahan University of Medical Sciences, Isfahan, Iran; 2 Student’s Research Committee, Shahrekord University of Medical Sciences, Shahrekord, Iran

Dear Editor,

We read with great interest the recent article by Wang et al. [1] entitled, “No seasonal variation in physical activity of Han Chinese living in Beijing”. In this study, the authors evaluated physical activity levels every two months across a complete year, while simultaneously assessed ambient temperatures and air pollution levels. They also investigated average hourly vector magnitude and percentage time spent at each physical activity. In this regard, 40 Han Chinese adults using GT3X accelerometers were recruited. Although this study was mostly a well-designed research, we have the following comments from a statistical point of view. In the statistical analysis section of the article, the authors stated that they used one way ANOVA to compare whether there were changes in body weight and body fitness between months. One way ANOVA is used for comparison of the means of more than two independent groups [2–4]. But the groups of this study are not independent, because they did not use different groups at each time points of evaluation (every two months). Indeed, they measured their variables in one group of 40 adults every two months across a complete year. Therefore, there were not independent groups in this study and they investigated one group at different time points. After assessment of the normal distribution of studied quantitative variables, the authors must use Repeated Measures ANOVA or Friedman test for comparison of the means of each variable at different times of measurement.

Taken together, we believe that most used statistical tests in this study and also results and discussion based on them are inappropriate and the author’s valuable study could better be used as citable clinical evidence if analyzed with appropriate statistical tests.
